# Supplementary material for: Tropical methane emissions explain large fraction of recent changes in global atmospheric methane growth rate
Source: Nat Commun. 2022 Mar 16;13:1378. doi: 10.1038/s41467-022-28989-z (PMC8927109; doi:10.1038/s41467-022-28989-z)
Supplement: Supplementary file 1 — Supplementary Information [file 41467_2022_28989_MOESM1_ESM.pdf]

Supplementary Information for "Tropical methane emissions explain large fraction of recent changes in global atmospheric methane growth rate"

Liang Feng[1,2], Paul I. Palmer[1,2]*, Sihong Zhu[3,2], Robert J. Parker[4,5], Yi Liu[3]

[1]National Centre for Earth Observation, University of Edinburgh, UK.

[2]School of GeoSciences, University of Edinburgh, UK.

[3]Institute of Atmospheric Physics, Chinese Academy of Sciences, Beijing, China

[4]National Centre for Earth Observation, University of Leicester, UK.

[5]School of Physics and Astronomy, University of Leicester, UK.

*Correspondence to: paul.palmer@.ed.ac.uk.

This PDF file includes:

Supplementary Text

Supplementary Figures 1-5

Supplementary Tables 1-3

References

Supplementary Text

A posteriori CH_4 and CO_2 Fluxes

Supplementary Table 1 reports annual mean *a priori* and *a posteriori* net CO_2 fluxes (PgC/yr) corresponding to the CH_4 emissions reported in Table 1 of the main text. For tropical emissions, we include contributions from three major TransCom-3 regions, including Tropical South America, Tropical Africa, and Tropical Asia.

Supplementary Figure 2 shows the monthly CO_2 and CH_4 fluxes during 2010-2019. Compared to the *a priori*, the *a posteriori* CO_2 flux shows more uptake during the boreal summer, resulting in smaller annual mean fluxes (Supplementary Table 1) and annual atmospheric CO_2 growth rates, consistent with NOAA observations. Supplementary Figure 2 also shows that *a posteriori* CH_4 emissions show higher peaks than *a priori* values, implying that the *a priori* inventories generally underestimate natural CH_4 emissions during the boreal summer.

Supplementary Table 2 compares the *a priori* and *a posteriori* annual CH_4 emissions from four tropical regions: Tropical South America, Tropical North Africa, Tropical Southeast Asia, and India (Figure 2). The *a posteriori* fluxes show much larger interannual flux variations than the corresponding *a priori* estimates, with their uncertainties typically 40% smaller than the *a priori* uncertainties.

Evaluation of *A posteriori* CH_4 and CO_2 Fluxes

Supplementary Figure 3 shows a summary comparison of TCCON and *a posteriori* model values of XCO_2 and XCH_4 from 2010 to 2019. *A posteriori* model values reproduce the spatial and temporal patterns of TCCON observations, with model bias for XCO_2 typically < 0.5 ppm and for XCH_4 within 10 ppb for most sites between 50°S to 50°N. At northern high latitudes (>50°N), the model shows a larger positive bias (15-20 ppb), which is due to the comparatively poor coverage by GOSAT and model errors. For sites between 50°S to 50°N the values of 1σ range between 5 and 20 ppb but typically < 10ppb. Over tropical continents (30°S to 30°N), the mean biases are typically smaller. However, there are only five sites over this region, with only a small number of sites with contiguous measurement coverage of more than a few years.

Supplementary Figure 4 shows the Theil-Sen slopes (da Silva et al, 2015) of *a posteriori* CH_4 fluxes, rainfall, skin temperature, and GRACE LWE from 2010 to 2019, inclusively. The main advantage of using the Theil-Sen estimator is to determine a long-term trend without being disproportionately influenced by short-term climate variations, e.g., 2015/2016 El Niño. The three main loci of positive CH_4 emission trends are over tropical Africa and tropical South America.

Over eastern tropical Africa, we find the increase in *a posteriori* CH_4 emissions (~0.4 g m^{-2} yr^{-1} per year) coincides with positive change in rainfall (up to 4 mm $month^{-1}$ per year) and LWE (up to 0.5 cm per year) and mainly focused over the Sudd and Lake Victoria, consistent with recent work (Lunt et al, 2019). We also find a negative surface temperature trend, which is consistent with an increase in wetland extend (Lunt et al., 2019). To the west (15°-25°E, 6°S-8°N) we also find a significant upward trend in emissions, driven by elevated rainfall during February-April in years after the recent El Niño. We find this increase is not significantly correlated with SST anomalies.

Over tropical South America, we find contrasting behaviour over the northeast and southwest of the continent. Over the northeast (mainly Colombia, Venezuela, Northeast Brazil and Guyana) we find a reduction in *a posteriori* CH_4 emissions corresponding to large drying trends that are reflected in the rainfall data and the GRACE LWE data. Similar drying trends have been reported for shorter periods by other studies (Marengo et al., 2016). In contrast, over southwest tropical South America (mainly Peru, Southwest Brazil and Bolivia), we find a significant increase in *a posteriori* CH_4 emissions that corresponds to a significant increase in rainfall and LWE.

Supplementary Figure 5 shows the spatial distribution of Pearson correlation coefficients between the seasonal CMAP rainfall data and three metrics we use to describe SST-based metrics over the Pacific, Atlantic, and Indian Oceans, including Niño3.4, Indian Ocean Dipole (IOD) and Pacific-Atlantic SST Dipole (PAD). The PAD is defined at the gradient between the tropical Atlantic (50°W-30°W, 5°N-20°N) and Pacific (120°W-90°W, 5°N-20°N) Oceans. We use the NOAA OI SST data to determine values for the IOD and PAD. Rainfall over the northeast and southwest Amazon (as defined above) are oppositely correlated with the Niño3.4 SST anomaly with maximal values of -0.6 and +0.5, respectively; we find a similar correlation pattern using the PAD. The contrasting responses of rainfall over the Tropical South America is due to changes in atmospheric circulation, specifically the Walker circulation, that are driven by SST anomalies (Wang et al.,

2006; Espinoza et al., 2014; Barichivich et al., 2018; Gloor et al., 2015). Rainfall over tropical East Africa is best described by the IOD, as expected (e.g., Finney et al., 2020; Wainwright et al., 2019).

We further investigate the relationship between local rainfall, temperature and CH_4 fluxes over the southwest and northeast Amazon and tropical East Africa, complementing the data reported in Table 2. We find the CH_4 flux is strongly correlated with rainfall over the three regions, with values from 0.4 to 0.8 (Supplementary Table 3). Over the dry tropics (e.g., northeast Africa and Northeast Amazon), we find negative correlations between -0.3 and -0.5 with temperature. Over wet tropical regions, we find that that influence of temperature is positive but weaker. Supplementary Table 4 is the corresponding version of Table 2 for tropical South America but using Niño 3.4 SST index instead of the Pacific-Atlantic dipole (PAD) SST metric (defined in the main text). We generally find that over our study regions, where CH_4 emissions have changed the most over our 2010-2019 study period (Supplementary Fig. 4), the PAD SST metric better describes variation in CH_4 fluxes than the Niño 3.4 SST index.

Supplementary Tables

Supplementary Table 1. Annual global and tropical terrestrial (i.e., TransCom=3 Tropical South America, North Africa, and Tropical Asia) net *a priori* and *a posteriori* CO_2 fluxes (PgC/yr) from 2010 to 2019, inclusively. Uncertainties denote the 1-σ value.

Year	Global CO_2 emissions (PgC/yr)		Tropical land (PgC/yr)	
	A priori	*A posteriori*	*A priori*	*A posteriori*
**2010**	8.6±1.0	4.8±0.4	2.7±0.3	1.5±0.2
**2011**	7.5±0.9	33±0.4	1.7±0.3	0.64±0.2
**2012**	8.5±1.0	5.4±0.4	1.3±0.3	1.2±0.2
**2013**	8.2±1.1	4.1±0.4	1.6±0.3	0.76±0.2
**2014**	8.1±1.1	4.4±0.4	1.9±0.3	0.67±0.2
**2015**	8.3±1.1	5.7±0.4	1.7±0.3	1.1±0.2
**2016**	8.6±1.1	5.4±0.4	2.1±0.4	1.5±0.2
**2017**	8.4±1.1	3.9±0.4	2.0±0.4	0.75±0.2
**2018**	8.6±1.0	4.7±0.4	2.0±0.3	0.63±0.2
**2019**	8.5±1.0	5.5±0.5	2,2±0.3	1.5±0.2

Supplementary Table 2. *A priori* and *a posteriori* annual CH_4 emissions and their corresponding uncertainties (in TgC/yr) from 2010 to 2019, inclusively, over Tropical South America, Tropical Africa, Tropical Southeast Asia and India (Figure 2).

	**Tropical South America**		**Tropical Africa**		**Tropical SE Asia**		**India**	
	A priori	*A posteriori*	*A priori*	*A posteriori*	*A priori*	*A posteriori*	*A priori*	*A posteriori*
**2010**	78.1±11.7	74.4±3.7	65.9±7.4	61.6±3.4	49.3±8.4	54.5±1.9	28.1±5.8	29.7±2.2
**2011**	73.7±11.3	79.4±3.7	63.8±7.3	58.3±3.2	47.7±8.2	56.3±1.9	28.4±5.8	31.2±2.2
**2012**	71.3±11.2	77.3±3.7	63.9±7.1	64.2±3.2	48.1±8.3	55.6±1.9	28.5±5.8	31.5±2.1
**2013**	73.4±11.2	77.3±3.6	63.2±7.4	62.4±3.2	48.4±8.3	55.0±1.9	28.7±5.8	30.4±2.2
**2014**	72.4±11.2	82.8±3.7	63.0±7.4	63.2±3.2	50.2±8.1	56.6±1.9	28.5±5.8	32.7±2.2
**2015**	70.2±11.1	83.8±3.7	62.2±7.6	71.1±3.4	52.6±8.6	55.0±1.9	28.6±5.8	34.6±2.2
**2016**	71.1±11.2	76.8±3.6	63.7±7.5	71.2±3.3	48.6±8.6	52.4±1.8	28.6±5.8	31.2±2.2
**2017**	70.4±11.2	83.1±3.8	63.7±7.4	67.9±3.3	47.8±8.2	53.6±1.8	28.7±5.8	33.9±2.2
**2018**	69.8±11.2	80.5±3.7	63.6±7.4	70.9±3.3	49.0±8.3	53.9±1.9	28.7±5.8	32.0±2.2
**2019**	70.6±11.2	85.2±3.7	63.4±7.4	73.3±3.5	53.2±8.5	52.5±1.8	28.7±5.8	32.4±2.2

Supplementary Table 3. Correlations between *a posteriori* CH_4 emission ($g/m^2/yr$) and CMAP rainfall (mm/month), and MERRA data products that describe soil moisture (unitless), and surface temperature (K) from 2010 to 2019, inclusively, over NE and SW tropical South America and tropical Africa. We have used a three-month smoothing window for the CH_4 emission to reduce the noise and to account for different time lags between CH_4 fluxes and environmental variables. Variables *n*, *r*, and *p* denote the number of data points, Pearson correlation coefficient, and the two-tail p-value.

	**Rain (mm/month)**<br>*n, r, p*	**Soil moisture (unitless)**<br>*n, r, p*	**Surface temperature (K)**<br>*n, r, p*
**NE tropical S. America**	120, 0.8, <0.001	120, 0.8, <0.001	120, -0.3, <0.001
**SW tropical S. America**	120, 0.7, <0.001	120, 0.6, <0.001	120, 0.3, <0.001
**East Africa**	120, 0.4, <0.001	120, 0.8, <0.001	120, -0.6, <0.001

Supplementary Table 4. Seasonal mean correlations between *a posteriori* CH_4 anomalies ($g/m^2/yr$) and the Niño 3.4 SST index from 2010 to 2019, inclusively, over NE and SW tropical South America. Variables n, r, and p denote the number of data points, Pearson correlation coefficient, and the two-tail p-value.

	**Wet season** n, r, p $\overline{DJFM}$	**Dry Season** n, r, p $\overline{JASO}$
**NE tropical S. America**	10, -0.5, 0.2	10, -0.7, 0.002
**SW tropical S. America**	10, 0.5, 0.1	10, 0.7, 0.02

Supplementary Figures

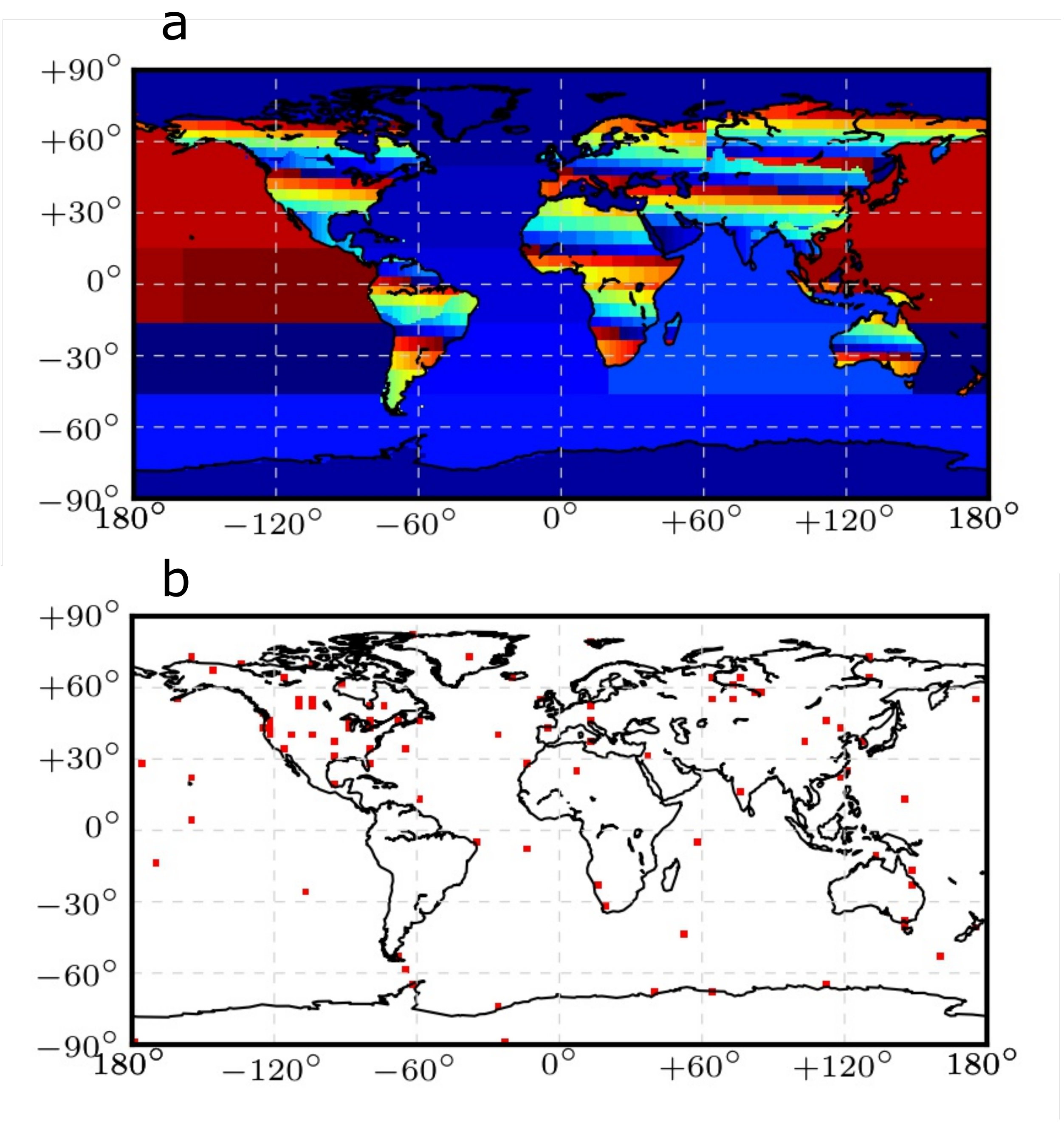

Supplementary Figure 1. **a** Basis functions that describe the 487 regions where we estimate CO_2 and CH_4 fluxes. Different colours are used to identify neighbouring sub-regions. **b** Distribution of *in situ* observations used to anchor our joint CO_2:CH_4 flux inversion.

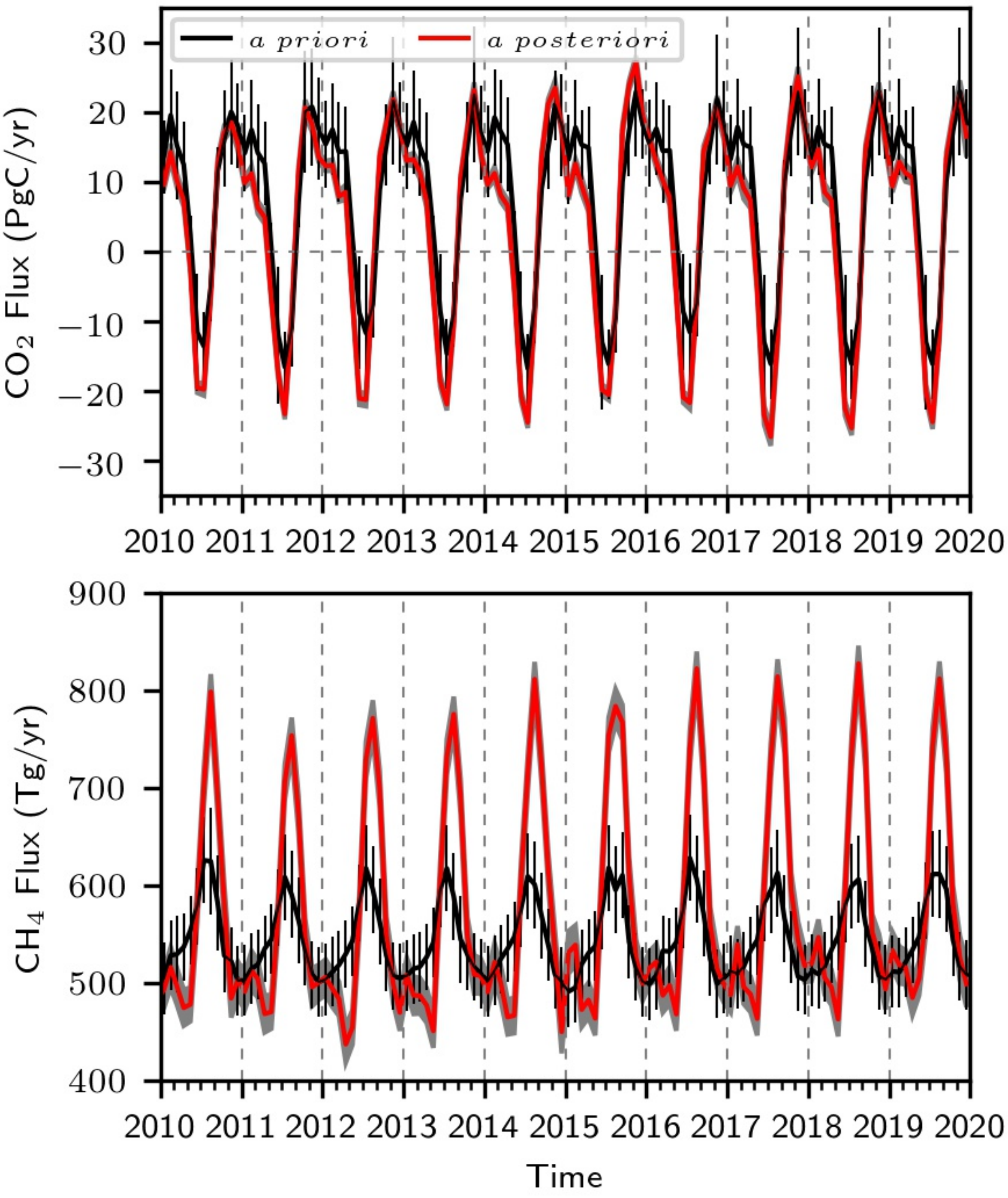

Supplementary Figure 2. Global monthly net (black) *a priori* and (red) *a posteriori* (top panel) CO_2 fluxes (PgC/yr) and (bottom panel) CH_4 emissions (Tg/yr) inferred from a simultaneous inversion of the GOSAT XCH_4:XCO_2 and NOAA *in situ* CO_2 and CH_4 data. Black vertical lines and the grey envelope denote the *a priori* and *a posteriori* 1σ values, respectively.

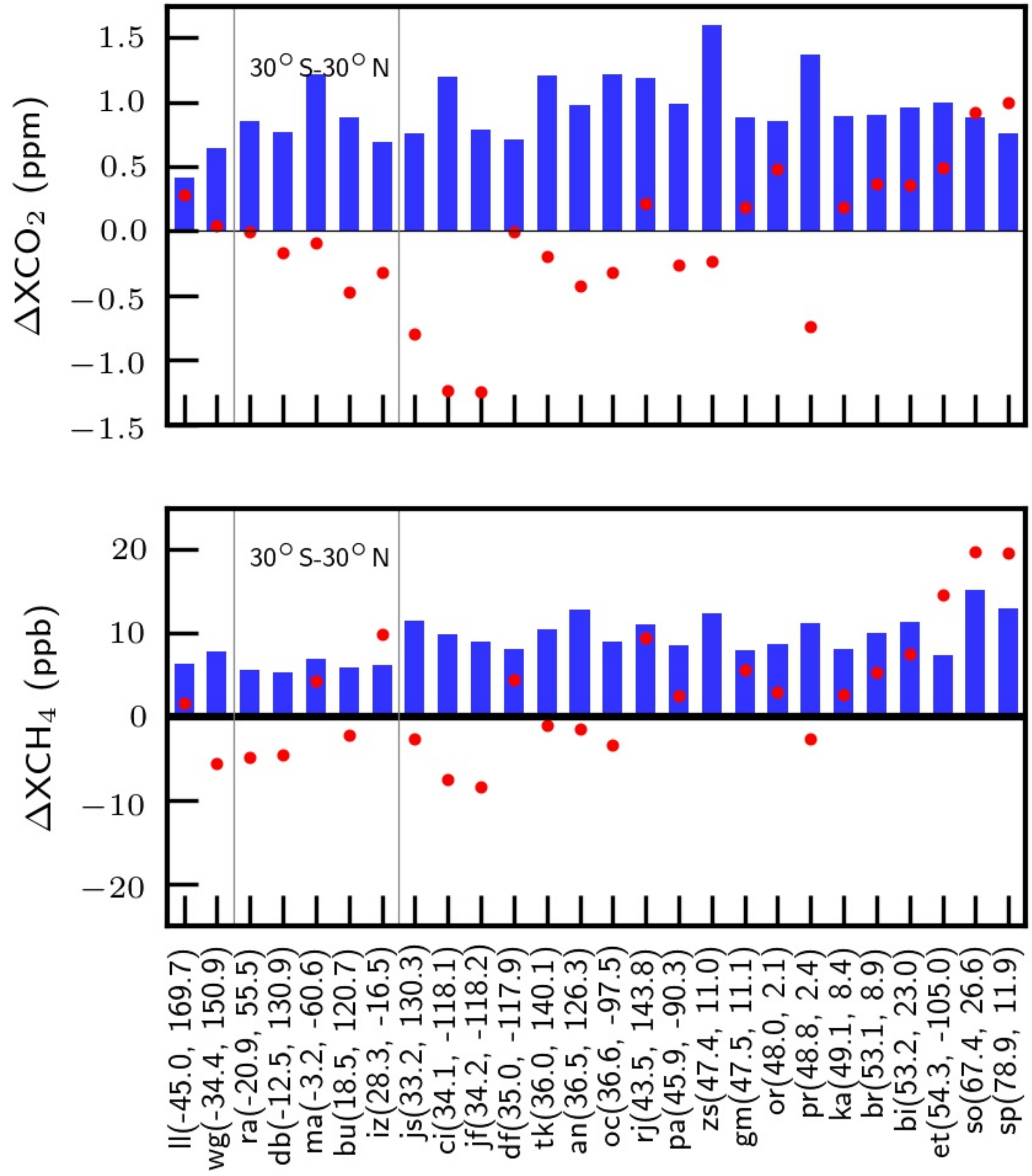

Supplementary Figure 3. Statistical comparison of *a posteriori* GEOS-Chem model and TCCON (version GGG2014) (top panel) XCO_2 and (ppm) (bottom panel) XCH_4 (ppb) values from 2010 to 2019. Red dots denote mean bias, and the blue bars denote the corresponding 1σ values. The grey vertical line denotes the sites that fall within ±30° latitude.

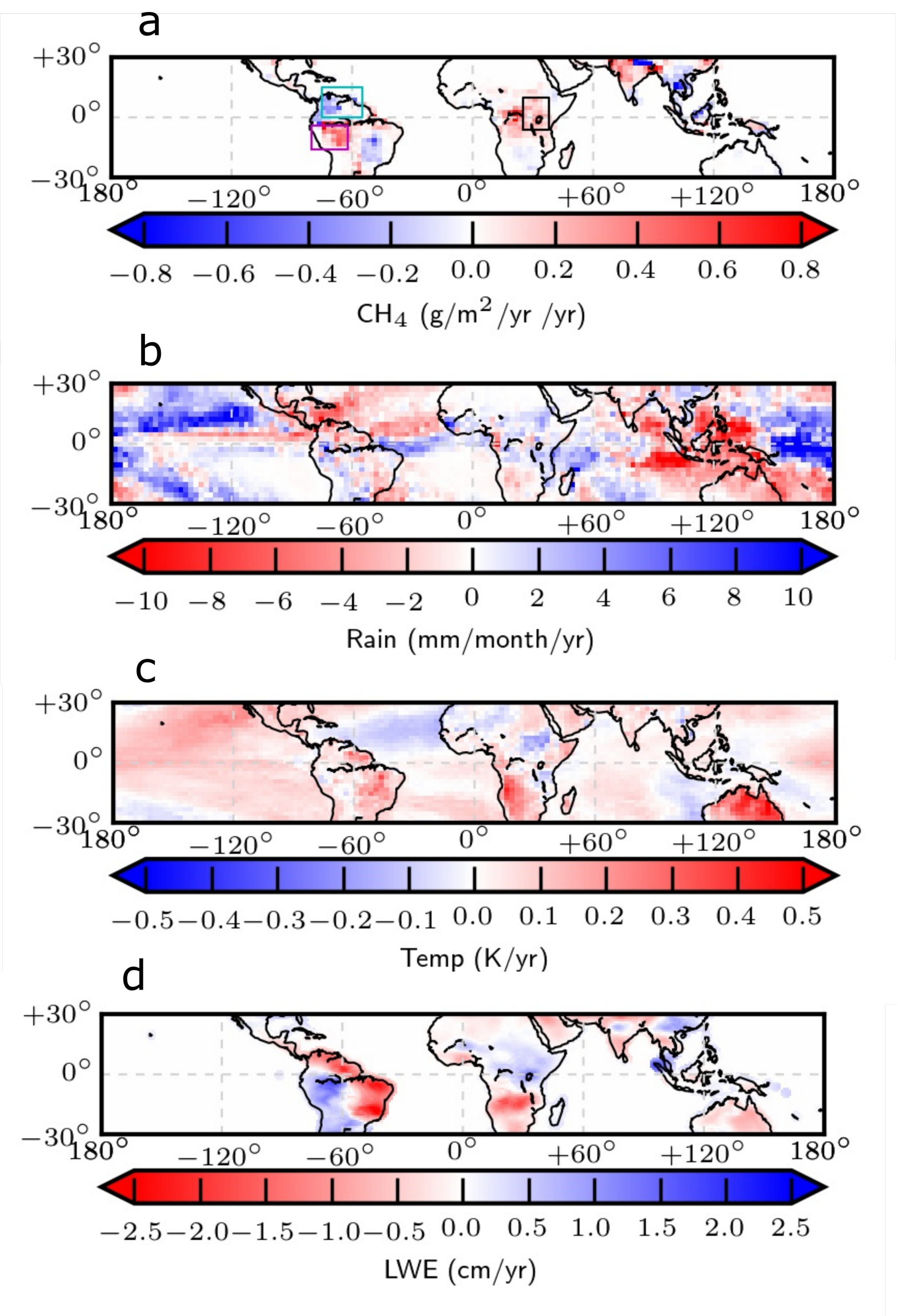

Supplementary Figure 4. Theil-Sen slopes determined from 2010 to 2019 for **a** CH_4 emissions ($g/m^2/yr\ yr^{-1}$), **b** CMAP rainfall ($mm/month\ yr^{-1}$), **c** MERRA2 skin temperature ($K\ yr^{-1}$), and **d** GRACE LWE ($cm\ yr^{-1}$, 2010-2017). We only show data that have a trend significantly different from zero ($p<0.05$). Coloured boxes in panel a denote three geographical regions where we report more detailed analysis.

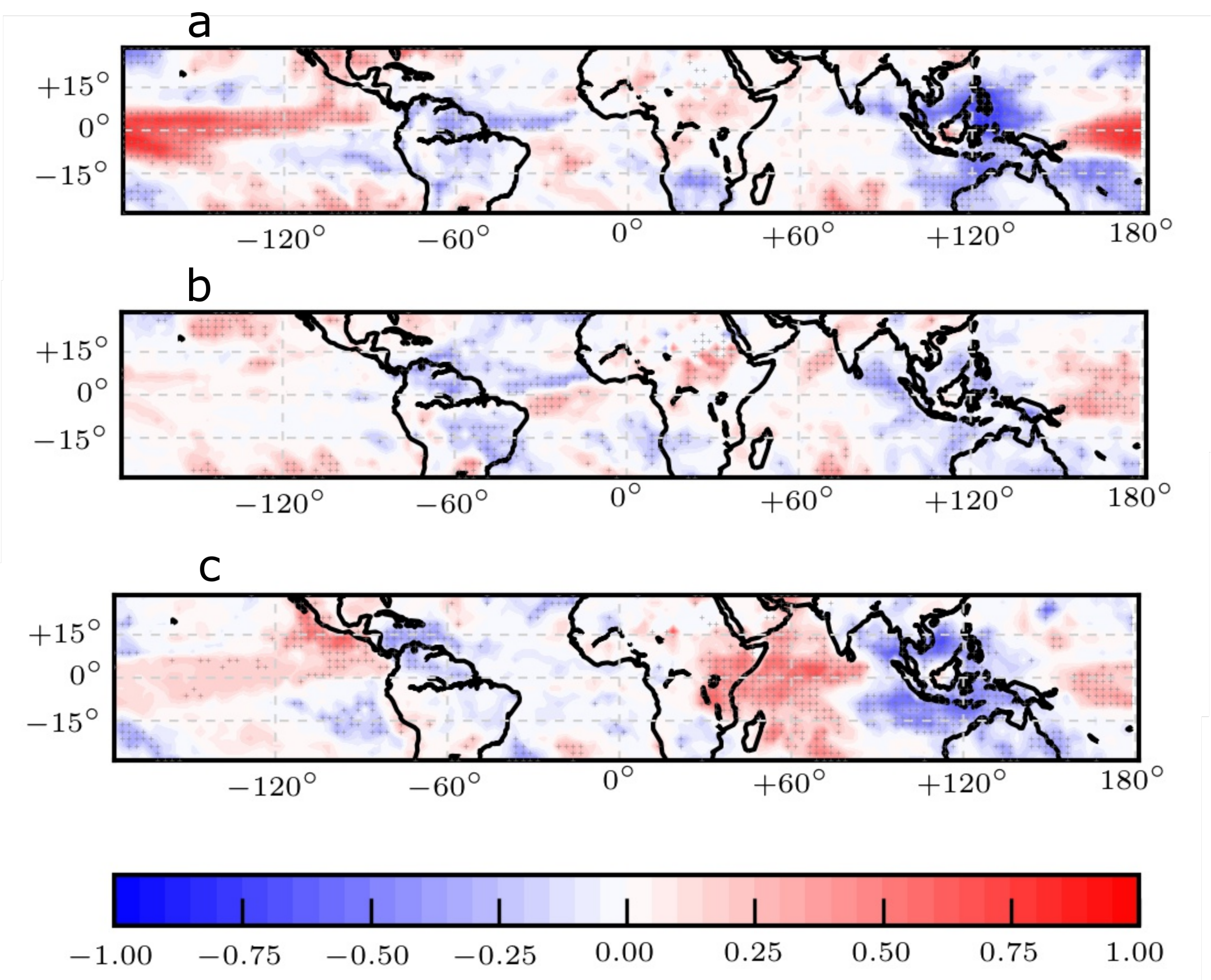

Supplementary Figure 5. Spatial distribution of Pearson correlation coefficients (R^2, unitless) from 2000 to 2019 between CMAP rainfall data and **a** Niño 3.4 index for Dec-March, **b** Pacific-Atlantic Dipole for Dec-March, and **c** Indian Ocean Dipole for Oct-Dec, which are determined from monthly NOAA SST values (defined in the Supplementary Material). Crosses denote regions with significant correlations ($p<0.05$).

Supplementary References

Barichivich J, Gloor E, Peylin P, Brienen RJW, Schöngart J, Espinoza JC, Pattnayak KC (2018): Recent intensification of Amazon flooding extremes driven by strengthened Walker circulation. *Sci Adv.*, doi: 10.1126/sciadv.aat8785, 2018

da Silva, R.M., Santos, C.A.G., Moreira, M. *et al.* Rainfall and river flow trends using Mann–Kendall and Sen's slope estimator statistical tests in the Cobres River basin. *Nat Hazards,* 77, 1205–1221 (2015). https://doi.org/10.1007/s11069-015-1644-7

Espinoza JC, Marengo JA, Ronchail J, Molina J, Noriega L, Guyot JL. 2014. The extreme 2014 flood in South-Western Amazon basin: the role of tropical-subtropical South Atlantic SST gradient. *Env. Res. Lett.* **9**: 124007, doi: 10.1088/1748-9326/9/12/124007.

Finney, D. L., Marsham, J. H., Walker, D. P., Birch, C. E., Woodhams, B. J., Jackson, L. S., and Hardy, S. The effect of westerlies on East African rainfall and the associated role of tropical cyclones and the Madden–Julian Oscillation. *Q. J. R. Meteorol. Soc.*, 146:727, 647-664, 2020.

Gloor M., Barichivich J., Ziv G., Brienen R., Schöngart J., Peylin P., Barcante Ladvocat Cintra B., Feldpausch T., Phillips O., Baker J., Recent Amazon climate as background for possible ongoing and future changes of Amazon humid forests. *Glob. Biogeochem. Cycl.,* 29, 1384–1399 (2015).

Lunt, M. F., Palmer, P. I., Feng, L., Taylor, C. M., Boesch, H., and Parker, R. J.: An increase in methane emissions from tropical Africa between 2010 and 2016 inferred from satellite data, *Atmos. Chem. Phys*., 19, 14721–14740, https://doi.org/10.5194/acp-19-14721-2019, 2019.

Marengo, J.A. and Espinoza, J.C: Extreme seasonal droughts and floods in Amazonia: causes, trends and impacts. Int. J. Climatol., 36: 1033-1050. doi:10.1002/joc.4420, 2016.

Wainwright, C.M., Marsham, J.H., Keane, R.J., Rowell, D.P., Finney, D.L., Black, E.and Allan, R.P. (2019): 'Eastern African Paradox' rainfall decline due to shorter not less intense Long Rains. *npj Climate and Atmospheric Science*, **2**(1), 1–9, https://doi.org/10.1038/s41612-019-0091-7.

Wang C. 2006. An overlooked feature of tropical climate: inter-Pacific-Atlantic variability. *Geophysical Research Letters* **33**: L12702, DOI: 10.1029/2006GL026324.
